# Extended compilation of autopsy-material measurements on lumbar ultimate compressive strength for deriving reference values in ergonomic work design: The Revised Dortmund Recommendations

**DOI:** 10.17179/excli2018-1206

**Published:** 2018-04-27

**Authors:** Matthias Jäger

**Affiliations:** 1 IfADo - Leibniz Research Centre for Working Environment and Human Factors, Ardeystr. 67, 44139 Dortmund, Germany

**Keywords:** lumbar spine, ultimate compressive strength, literature search, tolerance to compression, Revised Dortmund Recommendations, work design

## Abstract

Measures of human physical capacity are required in ergonomic work design. To avoid biomechanical low-back overload, criteria are needed to differentiate load and overload. With respect to the evaluation of manual materials handling and similar physical exposures regarding potential overload, the compression component of the forces transferred via lumbar discs or vertebrae are compared with the ultimate compressive strength of lumbar-spine segments in a common biomechanical approach. As mechanical load-bearing capacity cannot be quantified directly *in vivo*, forces are applied to dissected spinal elements up to failure, which is interpreted as a measure of ultimate strength or tolerance to compression. Corresponding autopsy-material measurements were collected from literature and examined regarding several conditions: At the very minimum, a specimen consists of a complete vertebra or a disc including the adjacent endplates; failure is identified definitely as lumbar; compressive-force application is quasi-static; results are given as single values etc. This study continues previous collations, the latest is dated on 2001 including 25 usable out of 47 investigations totally. Currently, 66 newly discovered seemingly appropriate studies were collected via a systematic literature search, 11 of them were added for subsequent analysis. Nearly 4,000 values were compiled, 1,192 remained for analysis. Human lumbar ultimate compressive strength varies between 0.6 and 15.6 kN, mean and standard deviation are 4.84 ± 2.50 kN. For data originating from donors of specified gender and aged 20 years or more, the distributions are characterised by 6.09 ± 2.69 kN for male adults (n=305) and 3.95 ± 1.79 kN for female adults (n=205). According to a linear regression model for donors aged 20 years or more, strength significantly decreases with age: 10.43 kN minus 0.923 kN per 10 years of age for males and 7.65 kN minus 0.685 kN per decade for females. Based on these gendered age relations, the “Revised Dortmund Recommendations” were derived ranging between 5.4 kN for males aged 20 years and 2.2 kN for males of 60 years or more. The corresponding recommended limits for females amount to 4.1 and 1.8 kN, respectively. A specific safety margin was implemented for young adults up to 25 years of age as skeletal strength may not be fully developed. Due to the compression-related and biomechanical nature of this approach, other influences like shear or torsion as well as psychological or psychosocial risk factors remain unconsidered despite their undoubted importance for initiating complaints, disorders and diseases at the low-back region.

## Introduction

The biomechanical load-bearing capacity of the lumbar spine is examined for a long time (e.g. Wyss and Ulrich, 1954[144]; Sonoda, 1962[132]), in particular, in the context of complaints, pain and diseases which affect frequently the low-back region including the spinal elements in the lower trunk section (e.g. Battevi et al., 2016[7]; Bergmann et al., 2017[9]). High mechanical load on the lumbar-spine structures like vertebrae and intervertebral discs results typically from activities like lifting or carrying heavy objects or manual patient handling (e.g. Chaffin, 1969[28]; Lavender et al., 2009[89]; Jäger et al., 2013[70]). In consequence, besides other influences like psychological, psychosocial, metabolic or genetic risk factors, physical work is interpreted a relevant reason for the initiation of low-back pain and the development of degenerative diseases (e.g. White and Panjabi, 1990[142]; Adams et al., 2002[1]; Kuiper et al., 1999[85]; da Costa and Vieira, 2010[33]). In its restricted nature of mechanical relations between load and load-bearing capacity in the human body, however, a biomechanical approach cannot explain the commonly multifactorial genesis of any low-back disorders, injuries, incidences, complaints and diseases (e.g. Hartmann et al., 2013[60]; Brinjikji et al., 2015[25][24]). Nevertheless, this approach is accepted as a valuable supplementary tool for the evaluation and design of manual handlings in ergonomics and occupational health (e.g. Kumar, 1999[86]; ISO, 2003[67], 2007[68], 2012[66]; Chaffin et al., 2006[29]; Hartmann et al., 2013[59]).

In the context of workload-overload associations, two general topics on dose-response relations can be differentiated which refer to short or long-term exposures. The latter issue became very prominent in Germany after the re-unification of the two German states in 1990 - the German Democratic Republic and the Federal Republic of Germany - as the possibilities and criteria for workers compensation with respect to severe mechanical low-back overload symptoms had to be harmonised. Through enacting a new regulatory directive “Intervertebral-disc related diseases of the lumbar spine caused by lifting or carrying heavy objects over many years or caused by activities in an extremely trunk-flexed posture over many years” as an “occupational disease” (BMA, 1992[14]), derivation of distinct criteria for assessing both individual lumbar disease expressions and individual loading profiles over the total occupational life became obviously necessary. Whereas literature review and discussions of a medical consensus group resulted in disease and functional deficit definitions (Bolm-Audorff et al., 2005[15]), the exposure-related criteria were specified regarding minimum values of object mass or exerted force, action frequency and total exposure duration (BMA, 1993[13]). Based on these specifications, cumulative dose models were configurated which consider each potential overloading action via the induced lumbosacral-disc compressive force and its duration and frequency applied (e.g. Hartung et al., 1999[61]; Jäger et al., 1999[76]). Meanwhile, this approach of a cumulative lumbar-load dose model is introduced in workers' compensation procedures in Germany. By means of the so-called German Spine Study EPILIFT (e.g. Seidler et al., 2009[128]; 2011[129]), a positive dose-response relation between the development of lumbar degenerative diseases, i.e. herniation or chondrosis accompanied by functional deficits, and occupational lumbar lifetime dose was found. Corresponding long-term studies focussing on low-back pain were provided by Lu et al. (2013[94]), Battevi et al. (2016[7]) and Bergmann et al. (2017[9]), also showing increased risks for higher physical workload.

Based on this knowledge on workload-overload associations at the lower back, an ergonomic work design shall be downright strived for in the preventing point of view. Thus, everyday working situations inducing “high” low-back load are identifyable and preventable via work design measures in future. However, specification of working situations resulting in “too high” low-back load needs overload criteria. With these aims, two benchmark manuals for the evaluation and design of handling tasks were provided, the socalled Work Practices Guide for Manual Lifting of the National Institute for Occupational Safety and Health (NIOSH, 1981[115]; Waters et al., 1993[138]) and “A Guide to Manual Materials Handling” (Mital et al., 1997[102]). In both guidebooks, besides psychophysical and physiological approaches, a biomechanical approach is recommended based on the comparison of the compressive forces acting in the lower spine with the ultimate compressive strength of dissected lumbar discs, vertebrae or larger segments. Hence, this *ex vivo* approach utilises unavoidably measurements on autopsy material due to the impossibility of *in vivo* determination of spinal structural strength. 

Considering the biomechanical idea of NIOSH (1981[115]) and Mital et al. (1997[102]), the provided biomechanical criteria - serving as compressive-force limits at the lumbosacral disc during a certain handling task - were analysed with regard to substantiation and reliability (e.g. Jäger and Luttmann, 1999[73]). However, autopsy-material measurement of lumbar ultimate compressive strength is usually related to a limited number of specimens in a certain examination (e.g. Wyss and Ulrich, 1954[144]; Nagel et al., 2013[114]). Hence, several literature compilation studies on lumbar static load-bearing capacity were performed (e.g. Genaidy et al., 1993[48]), in particular too, at author's institute in Dortmund according to changed demands or newly available literature (e.g. Jäger and Luttmann 1989[71], 1996[74]). 

The so-called Dortmund Recommendations were derived representing age-and-gender related limits for compressive forces at a lumbar disc or vertebra during manual materials handling to avoid biomechanical low-back overload, based on a multiple data amount compared to the samples in the aforementioned guidebooks (Jäger et al., 2001[77]). As shown in previous analyses, age and gender represent the strongest predictors for ultimate compressive strength (e.g. Jäger, 2001[69]). Other influences such as [i] donor's profession, [ii] health status and [iii] nutrition as well as test conditions like [iv] storage mode and [v] duration between section and experiment cannot be excluded, but could not be quantified. Further conditions serving as potential predictors are frequently not documented, on the one hand, and cannot be applied in preventive work design, on the other hand, such as body weight, specimen's cross-sectional area and the position of a test-induced, i.e. provoked damage within the lumbar spine ("lumbar level") between the thoracolumbar and lumbosacral discs. 

Due to progressed time, the aim of the current study is to provide the updated data sample, its analysis with respect to appropriateness and the newly derived so-called Revised Dortmund Recommendations. The compilation study at hand is embedded in a large cooperative project of the German Federal Institute for Occupational Safety and Health (BAuA) and the German Social Accident Insurance (DGUV) aiming to the development of a compendium of methods for risk assessment of physical workload (“MEGAPHYS”). 

## Methods

### Measurement of ultimate compressive strength in the studies considered

The load-bearing capacity of the lumbar spine was determined in numerous series on autopsy material dissected after death of the specimen donor. In such a measurement, the respective sample like a complete lumbar spine including the pelvis (e.g. Wyss and Ulrich, 1954[144]; Evans and Lissner, 1959[42]) or a so-called motion segment consisting of an intervertebral disc and its adjacent vertebrae (e.g. Brown et al., 1957[26]) is fixed in a testing machine. Axial compressive force is increased step by step or continuously in a quasi-static manner until a first maximum in a corresponding force-to-time or force-to-displacement curve is achieved. That means if the loaded specimen cannot fully withstand the applied force and is “yielding”, the force transferred via the specimen is diminished, and the force curve shows a steep dip. Thus, the resulting force value is termed yield or breaking point, failure or fracture load, strength, ultimate or static strength etc. The latter term contrasts to “dynamic strength”, if the applied force level is following a time-variant, mostly cyclic profile with a maximum lower than the ultimate strength. In ergonomic investigations, periodic force applications on spinal structures are common to examine fatigue fracture or failure load at vibration exposures (e.g. Brinckmann et al., 1988[19]; Huber et al., 2005[63]). Another dynamic loading profile is applied to investigate accidential impacts on spinal structures showing a very steep force increase (e.g. Plaue and Gesche, 1974[121]). Both dynamic load profiles are complementary to this paper's topic on (quasi-)static or ultimate strength. If provided in the cited reference, the slew rate ranges up to 2 kN/s in very most cases which corresponds to approximately 1 mm/s deformation velocity assuming a stiffness of about 2 kN/ mm (e.g. Panjabi and White, 1990[117]). This strength-test related criterion coincides with a quasi-static assumption compared to force's slew rate present in working life: Considering a load maximum of 6 kN achieved after about 1/4 second in a typical bi-manual floor-to-waist 20-kg lifting task (Jäger and Luttmann, 1989[71]), a slew rate of more than 20 kN/s will be adopted, that means it is much higher or “more dynamic” than most test conditions. In this regard, the study of Cheng et al. (1997[30]) applying the by far highest slew rate of our compilation sample of approximately 15 kN/s (8 mm/s) was not disregarded in principle, but interpreted as borderline. Due to missing data on donor's age and gender, however, the data of Cheng et al. remained non-considered in the derivation of recommended limits.

### Data compilation and selection

**Figure 1:**
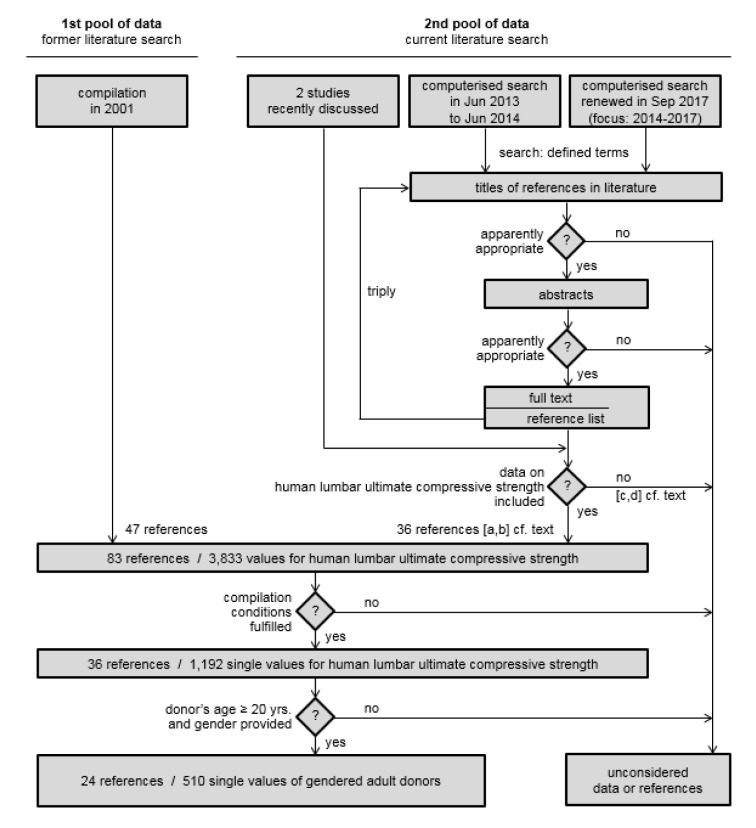
Flow chart of the current extended compilation of lumbar ultimate compressive strength data for deriving gender-specific age-related reference values to limit low-back biomechanical overload risk via ergonomic work design

Experimental findings on lumbar ultimate compressive strength were collected, examined with respect to the underlying aim and compiled based on two principles, as shown in the flow chart of Figure 1(Fig. 1). A first pool of data is represented by the results of the author's most recent compilation (Jäger et al., 2001[77]), and a second pool of newly available data was gathered via a systematic literature search. In an initial part, two studies recently discussed served as a starting point for further search (Huber et al., 2005[63]; Fischbeck, 2006[44]). The main search, however, had gone in computerised form via Scirus, Google Scholar and PubMed between June 2013 and June 2014 and was renewed in September 2017 via PubMed for the years 2014-2017. The search terms were compressive or compression, ultimate or maximum or maximal or static, failure or yielding or fracture or breaking load, low back or lumbar or spinal load or stress or strength or load-bearing capacity or tolerance as well as the respective German terms. Based on the title of the references provided in a collected source, seemingly relevant articles could be directly used for data collection or were used for analysing the included reference list with respect to further apparently appropriate studies. This procedure was repeated triply resulting in a total of 66 newly discovered studies which were categorised as follows: [a] 11 references providing appropriable values for human lumbar ultimate compressive strength, [b] 25 references providing values for human lumbar ultimate compressive strength, but these were evaluated inappropriate in the given context as, for example, a specimen's size was too small like a 1-cm cube or a test result was not attributable unequivocally to the lumbar section as the specimen was termed “thoracolumbar”, [c] 7 references without discrete data on ultimate strength, instead inter alia, showing prevalences of lumbar-spine fractures in specific populations, [d] 23 references providing inappropriate values as, for example, measured at an animal's specimen.

With respect to the size of a specimen matching this study's aim, the size ranges between a complete lumbar spine including the pelvis and sections of a lumbar spine. As a minimum enabling the transfer of the total force via a spine's cross-sectional area, an isolated vertebra or disc was considered, the latter including the endplates of the adjacent vertebrae. In consequence, results on lumbar ultimate compressive strength achieved at the spongy or cortical bone parts of a vertebra only (e.g. Weaver and Chambers, 1966[139]) were not included although those findings may be reasonable in prosthesis developments.

Due to missing details, in particular very early publications of Messerer (1880[101]), Lange (1902[88]), Göcke (1928[49]), Münchinger (1964[111]), Gozulov et al. (1966[50]), Krämer (1973[84]) and Morris (1973[104]) could not be examined adequately and remain disregarded in the subsequent description.

### Derivation of reference values

The procedure to derive reference values for the design of short-term manual materials handling jobs in the time window between a single task up to few working shifts follows the principle applied in the derivation of the so-called Dortmund Recommendations (Jäger et al., 2001[77]; ISO, 2012[66]). In a first step, literature data were gathered and analysed with respect to the study aim and conditions (i.e. human lumbar, compression mode, ultimate strength etc.). If age and gender of a specimen's donor were indicated and the donor was aged 20 years at minimum, gender-specific subgroups were established and strength regressions on age were calculated for both samples “adult males” or “adult females”. As a point on such a regression line represents a kind of mean value for given age and gender, the recommended limits were picked off from the regression lines and lowered by one standard deviation value of the respective subgroup in order to diminish the risk of strength overestimation. Recommendations are specified for every age decade between 20 years and above. For working persons of high ages, however, a specific deviation from this procedure was introduced: As lumbar ultimate compressive strength varies to a very small extent with age in this region, a unique compressive-force limit value is recommended for persons aged 60 years and more. 

Application of the former Dortmund Recommendations in ergonomic design practice over nearly two decades and numerous discussions lead to a further general modification, here for working persons of lower ages: To avoid misuse and misinterpretation of the newly derived Revised Dortmund Recommendations, a limit reduction for young adults aged 20 to 25 is preventively provided as the skeletal growth is possibly not terminated in those ages (e.g. Junghanns, 1979[80]; Krämer, 1994[83]).

## Results

### Lumbar ultimate compressive strength

#### Studies provided in the literature

Results regarding autopsy-material measurements on lumbar ultimate compressive strength are provided in the literature since many years. Based on a successive literature search by means of formerly found sources and the included references as well as on an intensive current literature examination (cf. Figure 1(Fig. 1)), 83 investigations in total were collated which presumably show, in part, considerable compressive-strength values for further utilisation in a limit derivation procedure. These studies are listed in Table 1 (References in Table 1: Adams & Hutton, 1982[2]; Adams et al., 1994[3]; Andresen et al., 1998[4]; Bartelink, 1957[5]; Bartley et al., 1966[6]; Bell et al., 1967[8]; Biggemann et al., 1988[10], 1991[11]; Bjarnason et al., 1996[12]; Bouxsein et al., 1996[16]; Brassow et al., 1982[18]; Brinckmann & Horst, 1983[21]; Brinckmann & Porter, 1994[23]; Brinckmann et al., 1986[22], 1988[19], 1989[20]; Brown et al., 1957[26]; Bürklein et al., 2001[27]; Cheng et al., 1997[30]; Cody et al., 1991[31]; Crone-Münzebrock et al., 1989[32]; Decoulx & Rieunau, 1958[34]; Dempster et al., 1993[35]; Ebbesen et al., 1999[37][36]; Edmondston et al., 1994[38], 1997[39]; Eie, 1966[40]; Eriksson et al., 1989[41]; Evans & Lissner, 1959[42]; Farfan, 1973[43]; Fischbeck, 2006[44]; Franke et al., 1976[45]; Galante et al., 1970[46]; Granhed et al., 1989[51]; Haidekker et al., 1999[52]; Hansson & Roos, 1981[53]; Hansson et al., 1980[54], 1987[57][56], 1988[55]; Hartman, 1974[58]; Hayes & Bouxsein, 1997[62]; Hutton & Adams, 1982[64]; Hutton et al., 1979[65]; Jayson et al., 1973[79]; Keller et al., 1989[81]; Konermann et al., 1999[82]; Lang et al., 1988[87]; Lin et al., 1978[90]; Link et al., 1997[91]; Lochmüller et al., 1998[93], 2002[92]; Martin et al., 1998[95]; McBroom et al., 1985[96]; McCubbrey et al., 1995[97]; Moro et al., 1995[103]; Mosekilde & Mosekilde, 1986[107], 1988[106], 1990[108]; Mosekilde et al., 1985[110], 1987[109], 1989[105]; Myers et al., 1994[112]; Nagel et al., 2013[114]; Ørtoft et al., 1993[116]; Perey, 1957[118]; Plaue & Gesche, 1974[121]; Plaue, 1972[120][119]; Porter et al., 1989[122]; Ranu, 1990[123]; Roaf, 1960[124]; Rolander & Blair, 1975[125]; Shirado et al., 1992[130]; Singer et al., 1995[131]; Sonoda, 1962[132]; Veenland et al., 1997[134]; Vesterby et al., 1991[135]; Waldt et al., 1999[136]; Weaver & Chalmers, 1966[139]; Werner, 1996[140]; Wyss & Ulrich, 1954[144]) chronologically and characterised by 83 reference numbers to enable a quick locating. 

The overview in Table 1(Tab. 1) demonstrates that only a few investigations became known in the fifties and sixties of the last century (in Table 1(Tab. 1), reference no. 1[144]: Wyss and Ulrich, 1954 to ref. no. 12[8]: Bell et al., 1967), whereas compressive strength was examined more often in the seventies and eighties (ref. no. 13[46] to 23[65] or no. 24[54] to 48[122]). The main proportion of studies come from the nineties (ref. no. 49[108] to 79[136]) and only a few could be found showing measurements performed in the 2000 millenium (ref. no. 80[27] to 83[114]). The intermediate three columns in Table 1(Tab. 1) illustrate the sample sizes: n_spec_ represents the number of specimens provided in a reference which were tested with respect to compressive strength, n_data_ corresponds to the amount of data which could be really found, and n_cons_ reflects the numbers of ultimate compressive force values where the underlying test conditions fulfill this study's assumptions so that those results were considered in the following analysis. In the column most right in Table 1(Tab. 1), the concrete reasons for disregarding a certain result are specified and exemplarily explained subsequently in correspondence to their consecutive mention. In case of two reasons or more for non-considering a certain strength-test result, the supplementary causes are given in parentheses (e.g. Plaue 1972, ref. no. 14[120]). 

As shown in Table 1(Tab. 1), Wyss and Ulrich (reference no. 1[144]) tested 8 specimens in total, however, one sample did not fail until achieving the top stop of the force-production measuring machine. That means, although intended, no structural damage could be provoked at the specimen due to equipment's limitation. By contrast, another specimen - a relatively long sample L1 to S1 - buckled laterally so that the corresponding failure-load value cannot be interpreted as ultimate compressive strength. Analysing reference no. 2[5], Bartelink (1957) mentioned that 10 specimens were tested, however, one strength value is provided only or, in other words, nine test results remain not shown. Similar is true regarding reference no. 7[124] (Roaf, 1960). The study of Perey (1957: no. 4[118]) considered totally 148 specimens, but three of them showed no damage and another three failed within the lowest throracic vertebra when samples spanning the thoracolumbar joint were tested (e.g. Th12-L1). Similar location-related cause for rejecting measurement results is true for the study of Waldt et al. (1999: ref. no 79[136]): the values cannot be clearly attributed to the lumbar section as “thoracolumbar” specimens consisting of six to eight vertebrae - including the intermediate discs - were tested and specification of the respective location is not provided.

Besides the aforementioned reasons for disregarding measurement results such as missing data, unclear damage location or disadvantageous test conditions, further specifics of studies led to non-consideration in the current compilation. For example, Sonoda (1962: ref. no. 8[132]) provides mean values for compressive strength at different lumbar levels without mentioning the sample sizes, so that the values are "weighted" and, in particular, of unknown weight. Bartley et al. (1966: ref. no. 9[6]) published maximum strain values, i.e. ultimate strength divided by the cross-sectional area. In the given context of deriving substantiated force limits instead of pressure or strain thresholds, such area-related values are insufficient. 

Despite of providing strength values “per area”, measurements achieved at parts of vertebrae were disregarded due to non fitting specimen size (e.g. Weaver and Chambers, 1966; Galante et al., 1970: ref. nos. 11[139] and 13[46]). In particular, the authors group of Mosekilde and coauthors (ref. nos. 31[110], 33[107], 36[109], 41[106], 47[105], 49[108]) performed numerous strength measurements on isolated cortical or trabecular material, however, the specimens' structure cannot reflect the mechanical behaviour of a complete vertebra due to its restricted size.

In few studies, strength was measured with regard to a temporal behaviour not coinciding with static or quasi-static conditions. For example, Plaue and Gesche (1974; ref. no. 19[121]) investigated impact loading, Brinckmann et al. (1986, 1988: ref. nos. 32[22], 38[19]) cyclic load on sub-ultimate levels. In other measurements, diverse mechanical prerequisites due to pre-damage were studied and compared to the strength behaviour of “normal” specimens. Those affected specimens were intentionally impaired mechanically prior to the subsequent test, or specimens showed metastatic signs in a “cancerous vertebral bone” (Lin et al., 1978; Crone-Münzebrock et al., 1989: ref. nos. 22[90], 43[32]). The corresponding strength-test results were disregarded. In other cases, ultimate compressive strength was derived indirectly, for example, by means of the priorily determined relation of strength to insufficiency fractures identified via quantititative computer tomography or via the association to bone mineral content (Hansson et al., 1987; Biggemann et al., 1991: ref. nos. 34[57], 51[11]). Then, the resultant “predicted” instead of measured strength values were rejected in the current context. 

A final group of disregard reasons is related to test results which were published more than once, but identified being the same (cf. “republished” or “republished later” in Table 1(Tab. 1), e.g. compare ref. nos. 24[54], 25[53] and 38[19], 42[20]), or which were not clearly identifyable or assignable in a provided diagram (ref. nos. 73[93], 74[95]). It is obvious that duplications should be also excluded as “undetectable” results cannot be included in the intended compilation. A specific remark refers to the studies of the Bouxsein authors group: Bouxsein et al. (1996: ref. no. 65[16]) and Hayes and Bouxsein (1997: ref. no. 69[62]) published strength-test results “adopted from Moro et al.” (1995: ref. no. 62[103]); in this reference, the underlying methodology is indeed described, but the respective data are missing.

In summary, 47 out of 83 publications contained not a single lumbar ultimate compressive strength value which was considered in the subsequently analysed compilation sample of 36 references. In total, putatively 6,046 specimens were tested, however, 3,833 data were provided in the respective descriptions of investigations only. Due to the reasons specified aforementioned - which were related to specimen or data conditions or to non fitting damage location or inappropriate loading type - 1,192 single values of 36 different examinations remained for further analysis.

### Data considered for further analysis

Characterised by 36 reference numbers, Table 2(Tab. 2) (References in Table 2: Adams & Hutton, 1982[2]; Adams et al., 1994[3]; Andresen et al., 1998[4]; Bartelink, 1957[5]; Bartley et al., 1966[6]; Bjarnason et al., 1996[12]; Brinckmann & Horst, 1983[21]; Brinckmann et al., 1986[22], 1988[19], 1989[20]; Brown et al., 1957[26]; Bürklein et al., 2001[27]; Cheng et al., 1997[30]; Crone-Münzebrock et al., 1989[32]; Decoulx & Rieunau, 1958[34]; Eie, 1966[40]; Eriksson et al., 1989[41]; Evans & Lissner, 1959[42]; Farfan, 1973[43]; Granhed et al., 1989[51]; Haidekker et al., 1999[52]; Hansson et al., 1980[54]; Hayes & Bouxsein, 1997[62]; Hutton & Adams, 1982[64]; Hutton et al., 1979[65]; Lin et al., 1978[90]; Lochmüller et al., 1998[93]; Myers et al., 1994[112]; Nagel et al., 2013[114]; Perey, 1957[118]; Porter et al., 1989[122]; Ranu, 1990[123]; Roaf, 1960[124]; Shirado et al., 1992[130]; Werner, 1996[140]; Wyss & Ulrich, 1954[144]) refers to the 36 references, which were published between 1954 (in Table 2(Tab. 2), ref. no. 1[144]: Wyss and Ulrich) and 2013 (ref. no. 36[114]: Nagel et al.) and which provide values for lumbar ultimate compressive strength fulfilling all stipulated test conditions, in particular, with respect to the specimen size. These 36 references represent a subgroup of the 83 references listed in Table 1(Tab. 1) (cf. Figure 1(Fig. 1), lower left). 

In the central columns of Table 2(Tab. 2), the number of specimens (n_cons1_, identical with n_cons_ of Table 1(Tab. 1)), mean and standard deviation (S.D.) are given for the respective subgroups. The latter is suppressed in case of one specimen per subgroup (cf. *: ref. nos. 2[5], 17[22]) and extra marked if a sample size is limited to 2 to 5 specimens only (cf. **: e.g. ref. no. 7[124], 8[40]). The highest number of considered data per reference amounts to 142 (ref. no. 4[118]: Perey, 1957), 11 of 36 references provide usable data of less than 10 values. Strength's mean values range between approximately 2.3 kN (ref. no. 29[140]: Werner, 1996) and 9.2 kN (ref. no. 23[122]: Porter et al., 1989), and S.D. values of the samples not indicated as very small vary between approximately 1.0 kN (ref. no. 34[52]: Haidekker et al., 1999) and 3.4 kN (ref. no. 28[12]: Bjarnason et al., 1996). Ranges of both means and standard deviations point to a large variation of the single values for strength. With respect to the total sample compiled from 36 references, lumbar ultimate compressive strength is 4.8 kN on average, and the overall standard deviation amounts to 2.5 kN based on nearly two thousand single values. 

Besides the numbers of strength values sketching roughly the respective test samples of the diverse authors (n_cons1_), further sample-amount numbers are listed in the column most right in Table 2(Tab. 2) (n_cons2_). These numbers correspond to results which were measured at specimens of donors of indicated gender and aging 20 years as a minimum. The respective strength values were subsequently used for the derivation of age-and-gender related maximum compressive-force limits in work design. As shown, n_cons2_ may fully reflect the total considerable number in the respective sample n_cons1_ (e.g. ref. nos. 1[144], 2[5], 13[54]), that means donor's age was always ≥20 years and donor's gender was specified throughout. In other cases, however, age and gender specifications were provided inconsistantly or only few times in a reference, such as in the studies of Hutton et al. (1979, ref. no. 12[65]: 51 of 58 results) or of Perey (1957, ref. no. 4[118]: 2 of 142 results). In twelve references, not a single lumbar ultimate compressive strength value could be subsequently considered as the presumed criteria regarding donor's age and gender were not fulfilled. From adult donors (≥ 20 years) indicated as female or male, 510 strength-test results were gathered in total (cf. Figure 1(Fig. 1), bottom left).

**Figure 2:**
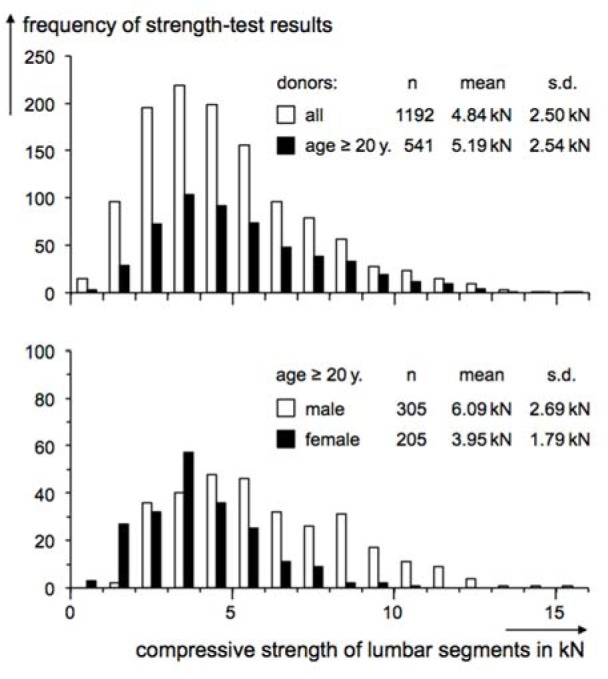
Frequency distributions of autopsy-material measurements on lumbar ultimate compressive strength compiled from literature; distributions regarding all donors of specified or unspecified gender and age (white above) or all donors of a specified age of 20 years or more, irrespective of gender (black above) or regarding male (white below) or female adults of these ages (black below)

Figure 2(Fig. 2) shows four frequency distributions with respect to lumbar ultimate compressive strength values in classes of 1 kN width. The diagram in the upper part corresponds to two “total samples”: the first refers to all values which were considered (n = 1,192) and are fulfilling the main methodologic conditions such as quasi-static and measured at a not too small specimen (cf. white columns). By contrast, the second distribution (cf. black columns) demonstrates the values of all donors of a minimal age of 20 years, irrespective of gender specification (n = 541). The diagram in the lower part contains the respective strength values for indicated donor gender, i.e. for totally 305 male or 205 female adults (in sum 510). Hence, 31 values were gathered from measurements on specimens of adult donors without gender specification.

Any of the four strength distributions in Figure 2(Fig. 2) demonstrates that ultimate strength spreads over a wide range which, however, differ among one another. Lowest values were found in the lowest class of up to 1 kN in the distributions of all donors (cf. white columns, diagram above), all adults (black above) and female adults (black below), but not for male adults (white below). With the exception of female adults (cf. black columns, lower diagram) showing the highest strength in the class “10 to 11 kN”, highest values reached unitarily up to 15 to 16 kN in the complementary three samples (all donors, all adults, male adults). Considering the statistical indicators of the four samples, mean and standard deviation values of the two total samples “all donors” and “all adults” differ noticeably less (cf. upper part) compared to the gender-specified pair of distributions (cf. lower part). Means' difference of the distributions showed above is about 7 % (4.84 vs. 5.19 kN) in contrast to 36 % related to the male-and-female sample pair (6.09 vs. 3.95 kN). Due to the similar width of the above distributions, S.D. values differ only marginally (2.50 to 2.54 kN). However, this contrasts to the distributions' difference in the lower part of Figure 2(Fig. 2): As varying over a distinctly wider range of strength in case of male donors compared to females, the male-related standard deviation is considerably larger (2.69 to 1.79 kN). Both differences are statistically significant (Student t-test, above p<0.01, below p<0.0001).

In summary, lumbar ultimate compressive strength measured at autopsy material shows a high variation. With respect to donor's gender, on average, mechanical load-bearing capacity is higher for males than for females - approximately 6 vs. 4 kN - and reaches up to approximately 16 or 11 kN, respectively.

### Age dependence

**Figure 3:**
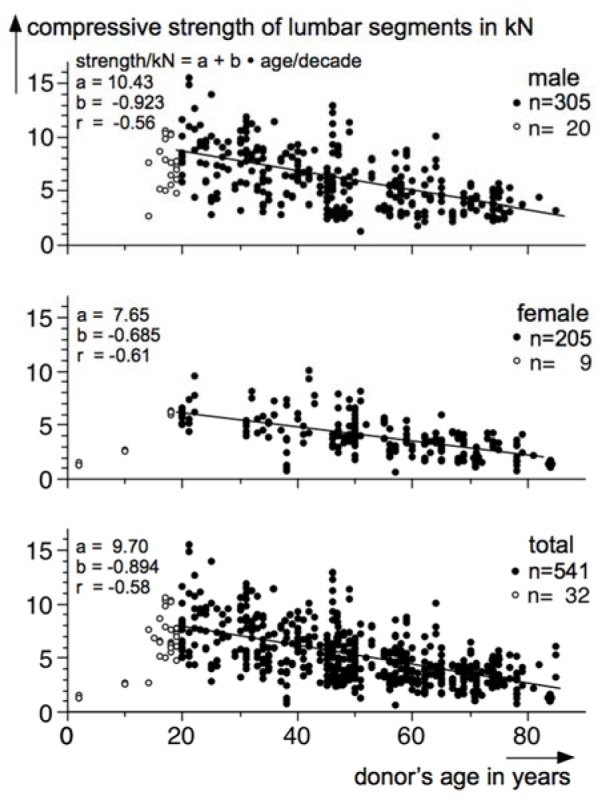
Relation of lumbar ultimate compressive strength on donor's age for all adults (bottom), female adults (middle) and male adults (top) based on the current data compilation of literature findings; linear regression modeling result in intercept (a), regression (b) and correlation coefficient (r) based on data of donor's age from 20 years (closed symbols) and neglecting lower-age data (open symbols)

The diagrams in Figure 2(Fig. 2) show wide distributions of the strength values. This is true even though age restrictions (≥ 20 years) and gender-related splitting are implemented. In correspondence to a common hypothesis (e.g. NIOSH, 1981[115]; Mital et al., 1997[102]), the newly compiled data samples were analysed with respect to donor's age. In this context, Figure 3(Fig. 3) demonstrates the dependences of ultimate compressive strength of dissected lumbar segments versus donor's age for three samples: total (bottom), female (middle) and male (top). Each dot represents a single strength-test result, the closed symbols correspond to a donor's age of 20 years or more and the open symbols to younger donors; the latter subgroups were not considered in the subsequent regression analyses. With respect to the lowest diagram, the unavoidable accumulation of values at higher ages - when death and, thus, section are more likely - becomes clearly apparent: many dots correspond to donors' ages around 50, 60 and 70 years, whereas quite less test results were gathered for ages between 20 and 40 years.

By means of the vertical scattering of the data, the point clouds in the three diagrams illustrate that ultimate strength varies not only versus age, but also for a distinct age. This finding can be attributed to “other” influences as mentioned in the introduction section, like donor's weight or specimen's cross-section. Neverthess, a clear age dependence is visible, and the scatter plots are declined for increasing age. This is supported by only few low-strength values up to 3 kN for low ages (20-30 years) and no high-strength values (> 6 kN) for high ages (> 70 years) in the diagrams. Corresponding linear regression analyses confirm this observation: The regression coefficient amounts to more than 0.9 kN per decade in case of male donors and nearly 0.7 kN for female donors, an intermediate value was calculated for the total sample of all adults irrespectively of gender (nearly 0.9). These “degressions” mean that lumbar ultimate compressive strength decreases by nearly 4 kN during 40 years at males in comparison to nearly 3 kN at females. According to the considerably higher ultimate strength of lumbar segments dissected from males than from females (cf. Figure 2(Fig. 2)), a higher intercept of the regression line for males was calculated than for females (approximately 10^1^/_2_ vs. 7^1^/_2_ kN), but - as aforementioned - strength is declining steeper with increasing age for males. Assuming an age of 25 years, averaged lumbar ultimate compressive strength amounts to approximately 8 kN in case of a male donor and nearly 6 kN for a female donor; corresponding predictions for donors aged 65 years are 4^1^/_2_ and 3 kN for males or females, respectively. The correlation coefficient varies between 0.56 and 0.61, and the regression models show statistically significant age dependences (slope ≠ 0, p<0.001).

In total, the results of regression analyses demonstrate that cadaver-related lumbar ultimate compressive strength show a clear dependence on donor's age. On average, mechanical load-bearing capacity is higher for younger adults than for older. The decrease over age is, furthermore, gender-specific and amounts to nearly 1 kN per 10 years of age for males and to about ^2^/_3_ kN per decade in case of females. 

### The Revised Dortmund Recommendations

#### Derivation of reference values

Application and compliance with reference values shall contribute to achieve the ergonomic preventive aim that the overload risk through manual materials handling and similar physical exposures is considerably diminished, even if a risk cannot be excluded completely. As the former “Dortmund Recommendations” do, the “Revised Dortmund Recommendations” are based on autopsy-material measurements on ultimate compressive strength of lumbar-spine segments. The wide scattering of data and the clear dependences to donor's age and gender suggest consideration of these predictors to enable a substantiated and adequate limit determination. According to the procedure described in the Methods section, the gendered linear regression functions of strength versus age served as the basis of limit derivation. In order to reduce overload risk, the recommended reference values reflect gender-specifically the regression line minus standard deviation. Deviations from this principle are implemented for young adults aged 20 to 25 years and seniors aged 60 years or more. 

The resultant suggested reference values representing gendered age-related limits for lumbar-spine compressive forces at manual handling of objects, subjects and other force exertion are provided in Table 3(Tab. 3). The recommended reference values vary between 4.1 and 1.8 kN for young or older female adults aged 20 or 60 years, respectively, and between 5.4 and 2.2 kN in case of males. 

#### Specifics

According to the correponding regression coefficents provided in Figure 3(Fig. 3), the limit differences are 0.7 or 0.6 kN per ten years for females, by contrast 1.0 or 0.9 kN for males - except for the decade 20 to 30 years of age. Regarding this exception, the limit differences amount only to 0.3 and 0.4 kN for young females or males due to the stipulated preventive limit reduction (cf. Methods section). That means the reference values for 20-years old females and males are precautionarily set to 4.1 or 5.4 kN, instead of 4.5 or 5.9 kN when strictly following the rule “regression line minus S.D.”. As mentioned in the Introduction section, this preventive action aims to reduce overload risk as spinal resistance capacity may not be fully developed at ages of 20 years and slightly more. 

Table 3(Tab. 3) shows, furthermore, that an unitary value is recommended for ages of 60 years or more: 1.8 kN for senior females and 2.2 kN for senior males. This recommendation results from specific regression analyses of strength versus age for gendered subgroups of donors' ages of [i] 50 years and beyond or [ii] from 60 or [iii] 70 years or more. A highly significant decrease was found in the first case for both genders (p<0.001), whereas a slope unequal zero was not verified for males in the 2nd and 3rd case (p>0.05). For females, however, statistical significance was found, even though on a low level. In consequence, no varying reference values were recommended for ages from 60 years and beyond, in particular, to pursue the aim of an unique procedure for both genders.

For persons younger than 20 years of age, no recommended compressive-force limits were derived due to the insufficient data amount of the underlying compiled sample: Only few compressive-strength values could be found in the literature, i.e. 9 in case of female adolescents and 20 in case of male ones (cf. open symbols in Figure 3(Fig. 3)). 

In summary, according to the large variation of lumbar ultimate compressive strength and according to the highly statistical significance of its age dependences for both genders, the newly derived “Revised Dortmund Recommendations” reflect these two influencing factors on individual capacity. These reference values are based on the current extended compilation of autopsy-material measurements on ultimate strength. In comparison with the formerly provided “Dortmund Recommendations”, the updated values deviate in 7 of 10 recommended limits for compressive forces acting in the lumbar spine during manual materials handling and biomechanically comparable exposures. For designing such physical work and following the biological fundamentals regarding lumbar-element's tolerance to compression, lower maximal lumbar load is recommended for older persons than for younger adults and - assuming identical age - lower limits should be applied for females than for males.

## Discussion

Aiming to a substantiated derivation of reference values serving as upper limits of forces in the lumbar spine induced by manual materials handling and similar physical exposures, results of autopsy-material measurements on ultimate compressive strength provided in the literature were collated and analysed with respect to several stipulated test conditions. This “extended compilation” follows the principles of previously published data collections and is based mainly on the most recently perfomed analysis (Jäger et al., 2001[77]), supplemented by newly available results of considerable amount. The number of seemingly reasonable investigations, i.e. literature references increased from 47 to 83, the number of examined specimens - as mentioned in the respective reference - gained from 2,517 to 6,046, the number of useful data in principle rose from 2,087 to 3,833, and the number of test results really included in the respective collation accrued from 776 to 1,192 single values for ultimate compressive strength of human lumbar-spine segments. By contrast, the number of test results considered in the procedure of limit derivation increased to a remarkably smaller extent: from 196 to 205 values in case of females and from 275 to 305 for males. As a consequence, the newly derived “Revised Dortmund Recommendations” should follow the updated gender-specific age-dependences and variances, on the one hand. However, they represent reference values for lumbar compressive forces of slight modification only, on the other hand, compared to the previously provided “Dortmund Recommendations”. Formerly ranging between 1.8 and 4.4 kN in case of females, reference values between 1.8 kN for older and 4.1 kN for young women are recommended due to the revision. The corresponding limit ranges differ to a larger extent in case of males: currently 2.2 to 5.4 kN instead of 2.3 to 6.0 kN before. With respect to recommended limits for ages of 30, 40 and 50 years, the modifications are kept within 0.1 kN for both genders. Although the differences between revised and original recommendations may be interpreted of minor extent, nevertheless, the renewed values are based on a considerably extended compilation, provide a larger “safety margin” for young adults and should be prioritised, therefore.

### Criticism of the method

The study at hand aims to generate substantiated support for a manifest approach common in ergonomics, namely to compare the load on the spine with its (bio-)mechanical load-bearing capacity. Except few direct, invasive measurements of intradiscal pressures (e.g. Nachemson and Morris, 1964[113]; Sato et al. 1999[127]; Wilke et al., 1999[143]), indicators of mechanical load like compressive forces at a lumbar disc are usually predicted applying an indirect methodology, that means via biomechanical model calculations (e.g. Bradford and Spurling, 1945[17]; McGill, 1992[98]; Mehta and Tewari, 2015[100]; Weston et al., 2018[141]). Similar is true for quantifying mechanical strength, since a direct measurement such as performed in mechanical structural analyses in material testing cannot be applied to living humans due to its non-destructive nature. Indirect fracture-load determination via bone mineral content (Hansson et al., 1980[54]), ash density (Mosekilde et al., 1989[105]), dual-energy x-ray absorptiometry or quantitative computed tomography (Lochmüller et al., 1998[93]) were examined, but had to be verified applying a reference method. Thus, measurements on spinal material dissected after death were performed and, in the current paper, analysed in order to achieve a helpful surrogate indicator of real-life load-bearing capacity. This problem of applicable or inapplicable methodologies at human beings may be interpreted unfortunate from the scientific point of view, however, mechanical-strength measurements on cadaveric specimens seem a possible, even reasonable and more or less unavoidable solution under the given restricted circumstances. If applying such a discussible methodology, nevertheless, its basis should be defined as trustworthy as possible. In this context, the former recommendations were examined after nearly two decades of application and modified according to the currently extended data sample.

Considering ultimate, short-term strength instead of long-term load-bearing capacity represents a further critical issue in the evaluation of lumbar-spine exposures with respect to potential overload. In a long-lasting dose-response approach, the numerous loadings during an occupational life are accumulated and related to the real incidences of disorders, complaints, injuries or similar. Comparison of cumulative doses may serve as a basis to derive thresholds of overload risk, and high-risk cumulative load can be specified on real data. Hence, such a retrospective approach should be prioritised from the scientific point of view. In contrast, short-term design measures reflect prospectively a hypothesis of overload prevention only - even if efficacy is verifiable via intervention studies or randomised controlled trials. Thus, the very opportunity of considerable risk reduction through implementation of substantiated short-term or even action-related design measures should be seized from the ergonomic, occupational medicine or ethical point of view. In consequence, compliance with reference values such as the Revised Dortmund Recommendations seems reasonable although they are derived from ultimate strength measurements.

The procedure of quantifying ultimate compressive strength is characterised by a monotonous increase of force until specimen's failure, whereas changing magnitudes of force on submaximal levels are applied to the human spinal structures in common life. In corresponding fatigue-strength measurements performed as well at autopsy material (e.g. Brinckmann et al., 1988[19]; Nagel et al., 2013[114]), specimen failure risk increases with increasing loading force and frequency. However, the number of cycles until failure in those investigations amount to several thousands only - 5,000 cycles in Brinckmann et al. and 20,000 cycles in Nagel et al. - which contradicts reality by a factor of ten or more: A typical working life provides few hundreds of loadings during a typical shift and thousands of days without such severe structural damages. In consequence, the applicability of the results of fatigue-related strength measurements on cadaveric specimens to the physiological conditions in a living human appears confined, and derivation of recommended limits with respect to loading magnitude and number of loading cycles seems awkward. To pursue such a target should prioritise, therefore, performance of epidemiologic studies. 

A further disadvantage of the current investigation into ultimate compressive strength may be seen in disregarding other indicators of mechanical load, such as torsional and bending moments of force and, in particular, shear forces. It is undoubted that those modes of biomechanical load occur usually in combined form and in diverse expressions: Two-handed lifting in front of the body induce mainly sagittal shear and compressive forces at the lumbar discs, whereas handling tasks like one-handed bricklaying show considerable “asymmetric” load characterised by lateral shear force and lateral bending moments. However, corresponding strength tests are rare compared to compression-related measurements (Jäger and Luttmann, 1992[75]) so that the derivation of tolerance limits seems more delicate and less substantiated. Nevertheless, such recommendations for work design are available with respect to moments (Tichauer, 1978[133]) and shear forces (e.g. McGill et al., 1998[99]; Gallagher and Marras, 2012[47]).

Recommended limits are also available from literature with respect to lumbar compressive forces. In the well-known “Work Practices Guide for Manual Lifting” (NIOSH, 1981[115]) and subsequent modifications (Waters et al., 1993[138], 1994[137]), an “Action Limit” of 3.4 kN acting at the lumbosacral disc is provided. In their “Guide to Manual Materials Handling”, by contrast, Mital et al. (1997[102]) recommended 3.9 kN for males and 2.7 kN as a limit for work design, which would represent nearly 70 percent of the mean ultimate compressive strength derived from a previous analysis of Jäger and Luttmann (1991[72]). However, those single limits “will not necessarily be protective for most individuals over 50 years of age or other susceptible persons” (NIOSH, 1981[115]) so that lower limits should be regarded, in particular, for older and/or female workforce. With respect to Mital et al. (1997[102]), a substantial part of the population remains unprotected too, namely those persons showing a lumbar compressive strength of up to 70 percent of average. According to this paper's compilation with means of 6.1 and 4.0 kN for males or females, respectively, a proportion of 70 percent amounts to 4.3 or 2.8 kN. Based on the current strength distributions sketched in Figure 2(Fig. 2) (lower part), approximately 30 percent of the values are lower than this criterion “70 percent of average” for both genders. Furthermore, the limits of both guidebooks took into account only a fraction of available data on ultimate compressive strength and must be evaluated being less substantiated than the current compilation: NIOSH considered two investigations for deriving its biomechanical criterion, which at best is weakly justified by the cited results (Jäger and Luttmann, 1999[73]); Mital et al.[102] referred to six investigations besides our summarisation. Therefore, the current data collection and examination provide a remarkable extension of analysed findings on compressive load-bearing capacity of the lumbar spine and is interpreted a more comprehensive basis for a substantiated derivation of reference values to diminish overload risk.

Obviously, compliance with single limit values as recommended by NIOSH (1981[115]) or Mital et al. (1997[102]) is easier to be achieved than application of gendered age-related reference values like the Revised Dortmund Recommendations. However, the latter recommendations provide a higher grade of overload protection for females and/or older working persons and should therefore be prioritised. In contrast, the Dortmund limits for younger persons are chosen higher than those of the guidebooks and could be questioned. As lower limits for younger adults are not substantiated by the underlying strength-test results and by the “biologic reality“ of higher load-bearing capacity for younger adults than for older ones, lower reference values would be contentious, detrimental to credibility and, in the long run, adversely effecting the acceptance in ergonomic practice. 

In most statistical tests, independence of the elements of a sample among one another is postulated a prerequisite prior to testing (e.g. Sachs, 1984[126]). If several specimens are dissected from a single donor and all strength-test results are regarded in a “specimen-related analysis”, this statistical requirement is not fully met. This is also true in the current compilation study. However, due to the applied selection criterion of minimal size, the average number of specimens taken from a single donor is very small (approximately 2) in comparison with the total number of specimens (305 for male, 205 for female adult donors). In a previous compilation, “donor-related analysis” was therefore performed when only one randomly chosen strength-test result per donor was considered in the corresponding gendered regression analysis over age (Jäger et al., 1991[78]). The hypothesis of statistical difference between specimen- and donor-related analysis was clearly falsified, i.e. intra-individual differences are lower than the inter-individual variation. Hence, the current study comprises all strength-test results fulfilling the predefined methodological requirements.

## Conclusions

In ergonomic work design of manual materials handling and similar physical exposures, measures of human physical capacity are needed to differentiate load and overload. Serving as substantiated upper limits of compressive forces acting at the lumbar spine, the Revised Dortmund Recommendations represent an easily applicable tool to evaluate handling tasks regarding biomechanical overload in a time window of a shift up to several shifts. For long-term exposure assessments, however, cumulative dose models should be prioritised. The Revised Dortmund Recommendations, derived from the current extended compilation on autopsy-material measurements on human lumbar ultimate compressive strength, consider the biological fundamentals of gender-specific age-related load-bearing capacity and specify lumbar-spine's tolerance to compression. So, identification of too high low-back exposures is enabled, in particular, for susceptible persons like older and/or female working persons. A comprehensive analysis of work-induced stress and strain should not be restricted to disc-compressive load only and should imply further aspects besides biomechanics such as epidemiological, physiological, psychological and psychophysical approaches.

## Acknowledgements

Grant: DGUV project No. 617.0-FP-0358 B; BAuA project No. F2333

Development of a compendium of methods for analysing the risks of physical exposures - A cooperative project of the German Federal Institute for Occupational Safety and Health (BAuA) and the German Social Accident Insurance (DGUV); 

Part A: Risk assessment regarding the musculoskeletal systems - Enhancing and evaluating methods and tools for analysing the risks of physical exposures in terms of the biomechanical effects on the musculoskeletal systems and, in particular, the spine.

Special thanks are due to the following persons: B. Hartmann (Hamburg, Germany) for encouraging to update the literature compilation, A. Luttmann (Dortmund, Germany) for long-term and specific scientific support, K. Lukaszewski (Dortmund) for accurate and careful preparation of tables and figures, K. Kostarelos (Dortmund) for engaged support to the literature search.

## Conflict of interest

The author declares that he has no conflict of interest.

## Figures and Tables

**Table 1:**
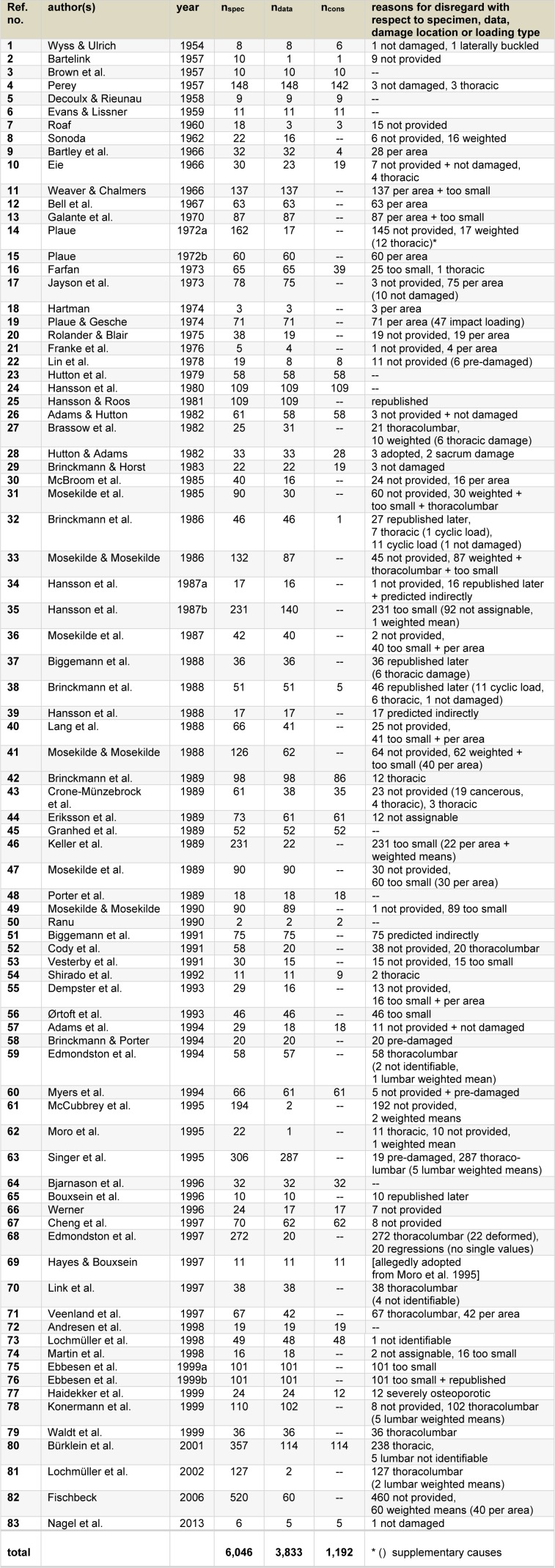
Overview on investigations into lumbar ultimate compressive strength of autopsy material as collated from the literature. With regard to the respective study: number of tested specimens (n_spec_), number of provided data (n_data_), number of test results considered in the current compilation (n_cons_) and reasons for disregarding specified results

**Table 2:**
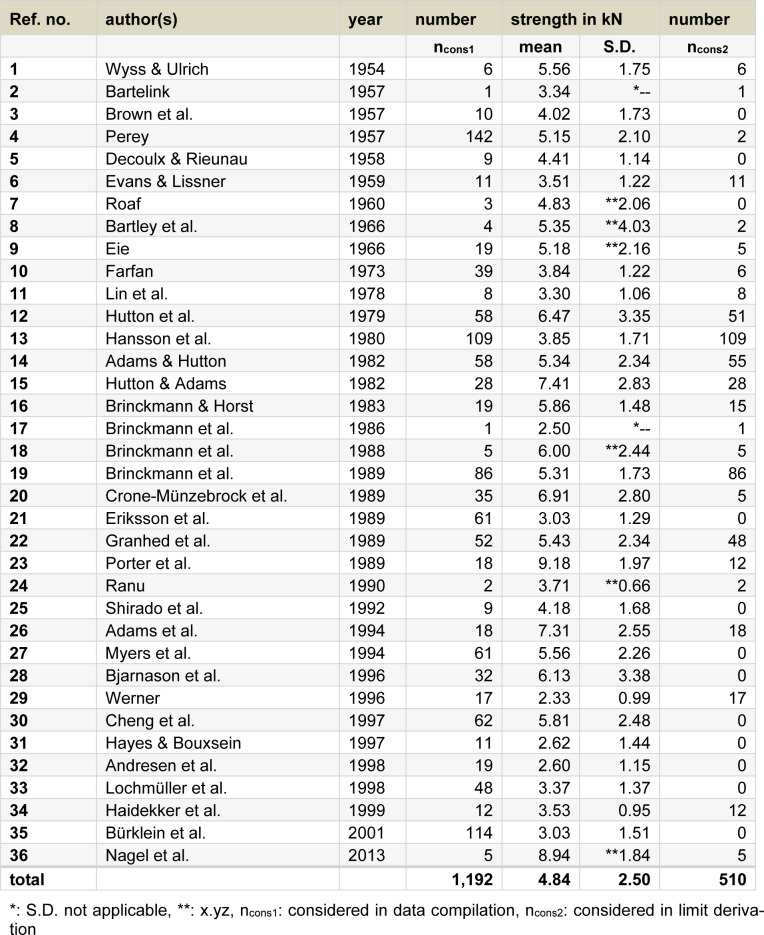
Investigations into and data on lumbar ultimate compressive strength of autopsy material as collated from the literature. With regard to the respective study: number of test results considered in the current compilation (n_cons1_), strength's mean and standard deviation (S.D.) and (cf. right column) number of test results considered in the subsequent derivation of reference values (n_cons2_)

**Table 3:**
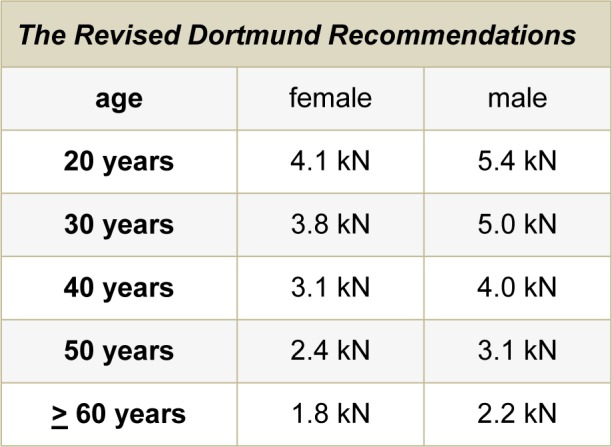
The “Revised Dortmund Recommendations” based on the current extended compilation of autopsy-material measurements on lumbar ultimate compressive strength and to be applied in work design analyses: Gender-specific age-related reference values for maximum lumbar compressive forces during manual materials handling and similar physical exposures owing to diminish biomechanical low-back overload risk
